# Neuropeptide S receptor 1 (NPSR1) activates cancer-related pathways and is widely expressed in neuroendocrine tumors

**DOI:** 10.1007/s00428-014-1602-x

**Published:** 2014-06-12

**Authors:** V. Pulkkinen, S. Ezer, L. Sundman, J. Hagström, S. Remes, C. Söderhäll, G. Dario, C. Haglund, J. Kere, J. Arola

**Affiliations:** 1 Pulmonary Division, Department of Medicine, University of Helsinki, Helsinki, Finland; 2 Research Programs Unit, Program for Molecular Neurology, University of Helsinki, and Folkhälsan Institute of Genetics, Helsinki, Finland; 3 Department of Pathology and Oral Pathology, Haartman Institute, University of Helsinki and HUSLAB, Helsinki, Finland; 4 Department of Pathology, Haartman Institute, University of Helsinki and HUSLAB, Haartmaninkatu, Helsinki, Finland; 5 Department of Biosciences and Nutrition, Karolinska Institute, Huddinge, Sweden; 6 Systems toxicology, Finnish Institute of Occupational Health, Helsinki, Finland; 7 Department of Surgery, Helsinki University Hospital and University of Helsinki, Helsinki, Finland; 8 Research Programs Unit, Translational Cancer Biology, University of Helsinki, Helsinki, Finland

**Keywords:** Neuropeptide S, Neuroendocrine tumor, Neuroendocrine marker, Immunohistochemistry

## Abstract

Neuroendocrine tumors (NETs) arise from disseminated neuroendocrine cells and express general and specific neuroendocrine markers. Neuropeptide S receptor 1 (NPSR1) is expressed in neuroendocrine cells and its ligand neuropeptide S (NPS) affects cell proliferation. Our aim was to study whether NPS/NPSR1 could be used as a biomarker for neuroendocrine neoplasms and to identify the gene pathways affected by NPS/NPSR1. We collected a cohort of NETs comprised of 91 samples from endocrine glands, digestive tract, skin, and lung. Tumor type was validated by immunostaining of chromogranin-A and synaptophysin expression and tumor grade was analyzed by Ki-67 proliferation index. NPS and NPSR1 expression was quantified by immunohistochemistry using polyclonal antibodies against NPS and monoclonal antibodies against the amino-terminus and carboxy-terminus of NPSR1 isoform A (NPSR1-A). The effects of NPS on downstream signaling were studied in a human SH-SY5Y neuroblastoma cell line which overexpresses NPSR1-A and is of neuroendocrine origin. NPSR1 and NPS were expressed in most NET tissues, with the exception of adrenal pheochromocytomas in which NPS/NPSR1 immunoreactivity was very low. Transcriptome analysis of NPSR1-A overexpressing cells revealed that mitogen-activated protein kinase (MAPK) pathways, circadian activity, focal adhesion, transforming growth factor beta, and cytokine–cytokine interactions were the most altered gene pathways after NPS stimulation. Our results show that NETs are a source of NPS and NPSR1, and that NPS affects cancer-related pathways.

**Electronic supplementary material:**

The online version of this article (doi:10.1007/s00428-014-1602-x) contains supplementary material, which is available to authorized users.

## Introduction

*Neuropeptide S receptor 1* (*NPSR1* previously known as *GPRA* and *GPR154*) is a G protein-coupled receptor that induces intracellular signaling upon stimulation by neuropeptide S (NPS) via mobilization of calcium, increased cyclic adenosine monophospate (cAMP) levels, and activation of the mitogen-activated protein kinase (MAPK) pathway [1, 2]. *NPSR1* encodes several splice variants in humans, but only two full-length variants with unique intracellular carboxy-termini, NPSR1-A and NPSR1-B, are expressed on the cell surface [2]. NPSR1 is mostly expressed in the central nervous system [3], but also in specific peripheral cell types, such as monocytes/macrophages [4–6] and neuroendocrine cells of the gut [7, 8]. The *NPSR1* locus has shown genetic associations with inflammatory diseases, such as asthma [9–16], inflammatory bowel disease [17], and rheumatoid arthritis [18, 19], as well as with anxiety [20] and various stress-related phenotypes [21–24]. NPS controls multiple neuroendocrine and behavioral responses, such as stress reactions in rodents [25–28]. In addition, NPS modulates cell growth of human Colo205 colon cancer cells [1] and porcine splenic lymphocytes [5]. In an NPSR1-A overexpressing human embryonic kidney epithelial cell line, NPS stimulation increased expression of genes that encode peptide hormones and neuropeptides secreted by enteroendocrine cells [7]. Transcriptome analyses revealed that NPSR1-A and NPSR1-B regulate essentially identical sets of genes, but the signaling effects were stronger with NPSR1-A [29, 30].

Neuroendocrine cells are distributed widely throughout the body as disseminated cells or glands. Tumors originating from neuroendocrine cells are rare [31]. They share uniform histological hallmarks, but in addition to morphology diagnosis of neuroendocrine tumors (NETs) is based on cell-specific markers that can be detected by immunohistochemistry (i.e., chromogranin-A and synaptophysin). In addition, the proliferation marker Ki-67 is essential for determining tumor grade and predicting prognosis for gastroenteropancreatic (GEP) NETs. New tumor specific and prognostic markers for the diagnosis of NETs are still needed.

Stress responses activate the neuroendocrine and sympathetic nervous system, and can impact on cancer development by immune dysregulation. Because the NPS/NPSR1 system acts on the hypothalamic–pituitary–adrenal (HPA) axis to affect stress response and has direct and indirect effects on immunity, we hypothesized that NPSR1 may have important effects on neuroendocrine neoplasms. To evaluate whether NPS/NPSR1 might be used as markers for certain NETs, we studied the expression of NPS and NPSR1 in neuroendocrine tumors from different organs. NET diagnosis was confirmed through chromogranin-A and synaptophysin immunostaining and NET grade was analyzed by Ki-67 proliferation index (PI). To characterize signaling pathways affected by NPS/NPSR1, we analyzed the effects of NPS on the global gene expression pattern of a human SH-SY5Y neuroblastoma cell line which overexpresses NPSR1-A and is of neuroendocrine origin. Our results show that NPS and NPSR1 are expressed in NETs, and NPS activates pathways important in cancer development.

## Materials and methods

### Tumor material

We collected 91 paraffin-embedded tissue samples of NET from rectum, ileum, skin, lung, thymus, appendix, parathyroid, thyroid, stomach, pancreas, liver metastasis, adrenal medulla, and extra adrenal ganglions (Table 1). Paraffin-embedded tissue samples from patients who underwent surgery for histologically confirmed colorectal cancer [32] were used to assess expression of NPSR1 in adenocarcinomas. The samples were collected at the Department of Pathology of HUSLAB and Haartman Institute, University of Helsinki. The study protocol has been approved by the Ethics Committee of Helsinki University Central Hospital (3990/04/046/07).

**Table 1:** Study material

Tissue	Histological diagnosis	Number	Male	Female	Age	PI ≤2 %	PI 3–20 %	PI >20 %
Stomach	Neuroendocrine neoplasm^a^	6	4	2	65	–	3	3
Ileum	Neuroendocrine neoplasm^a^	5	3	2	64	3	2	–
Appendix	Neuroendocrine neoplasm^a^	7	3	4	51	4	3	–
Rectum	Neuroendocrine neoplasm^a^	6	1	4	58	2	3	1
Pancreas	Neuroendocrine neoplasm^a^	13	4	9	50	4	8	1
Lung	Carcinoids, small cell carcinoma	14	4	10	59	4	3	7
Thymus	Neuroendocrine carcinoma	1	–	1	43	–	1	–
Thyroid	Carcinoma medullare	5	2	3	46	3	2	–
Parathyroid	Adenoma	5	1	4	58	4	1	–
Adrenal gland	Pheochromocytoma	5	3	2	56	2	3	–
Extra adrenal	Paraganglioma	5	2	3	69	3	2	–
Skin	Merkel cell carcinoma	4	3	1	77	–	–	4

^a^Distribution of proliferation index (PI) values proposed by WHO 2010 was used for grading of gastroenteropancreatic neuroendocrine tumors (GEP-NETs) to simplify the presentation of PI values. GEP-NETs were categorized into NET G1 tumors (PI ≤2 %), NET G2 tumors (PI = 3–20 %), and NEC G3 carcinomas (PI >20 %)

### Antibodies

For immunohistochemistry, mouse monoclonal antibodies against the synthetic peptide CREQRSQDSRMTFRERTER of the C-terminus of isoform A (NPSR1-A, amino acids 336–354) and against the synthetic peptide TEGSFDSSGTGQTLDSSPVA (NPSR1-N, amino acids 6–20) corresponding to the extracellular N-terminus of NPSR1 were used [7, 30]. Anti-mouse IgG antibodies (1:4,000,000, R0614, Vector) were used as an isotype control. Rabbit polyclonal anti-NPS antibodies (0.6 μg ml^−1^) were purchased from Abcam (Cambridge, UK). Rabbit IgG (1:500,000, R923, Vector) was used as a negative control. Antibodies against chromogranin-A (1:800) were purchased from Dako (Glostrup, Denmark) and Novocastra antibodies against synaptophysin (1:50) from Leica Biosystems. Chromogranin-A and synaptophysin expression were used to confirm the diagnosis.

### Tissue microarray construction and immunohistochemical techniques

Histopathological diagnosis was re-evaluated on hematoxylin–eosin (HE) sections by an endocrine pathologist (JA), who also selected representative areas of each tumor for construction of tissue microarray (TMA) blocks. One-millimeter-diameter punctures were taken from the borders and central area of each tumor with a semiautomatic tissue microarray instrument (Beecher Instruments, Silver Spring, MD, USA). Three parallel serial blocks were constructed, all including duplicate samples from each tumor.

For immunohistochemical analysis, 4 μm sections were cut from TMA blocks and placed on charged SuperFrost Plus slides (Thermo Scientific, Fremont CA, USA). After deparaffinization, tissue sections were pretreated in a pretreatment module (LabVision UK Ltd, UK) with Tris–HCl buffer pH 8.5 (NPSR1-A, NPSR1-N, chromogranin-A, synaptophysin) or Target retrieval solution (Dako) (NPS). Microwave antigen retrieval in Tris–EDTA pH 9.0 was used for Ki-67 antigen. After heat-mediated antigen retrieval, slides were cooled down to room temperature and treated with Peroxidase-Blocking Solution, Dako REAL (Dako). Immunohistochemical staining was performed with polymer detection kit EnVision^TM^ (Dako) in a LabVision Autostainer 480 (Thermo Scientific, Fremont CA, USA). The anti-NPSR1-A (1:3,000), NPSR1-N (1:400), and NPS (1:500) (Abcam, Cambridge, UK) antibodies were incubated for 1 h, and the Ki-67 antibody (1:200) (clone MIB-1, Dako) for 30 min at room temperature. The slides were counterstained with Mayer’s Hematoxylin (Dako) and mounted in aqueous mounting medium (Aquamount, BDH, Poole, UK).

The immunoreactivity of NPS as well as the staining for both N-terminus and C-terminus of NPSR1 was independently analyzed by two inspectors (LS, JH) using a scale of 0–3, and the consensus value representing the highest value of immunoreactivity in the six parallel spots was used for final analysis.

### Assessment of PI

PI was assessed by by image analysis software, ImmunoRatio [33, 34]. Image capture was performed by Nikon Eclipse 80i light microscope (×40 objective) connected to the Digital Sight DS-5 M (Nikon) digital camera and NIS-Elements F 3.0 image capture software. From every core biopsy, one digital image (JPEG format, Resolution 1,280 × 960) was captured from the most proliferative area of the tumor covering 50 % of the core biopsy. Using the ImmunoRatio’s Advanced Mode setting, threshold values for hematoxylin (−10) and DAB [10] were adjusted without correction equation. A blank field image was taken to balance uneven illuminations in the final digital images. Image analysis settings were kept the same after the light exposure (Manual exposure, 10 ms, Gain 1×) and thresholds were considered good. Of all the images available from each tumor, the highest PI was recorded.

### Cell culture

African green monkey kidney fibroblast cell line COS-7 was grown in Dulbecco’s modified Eagle’s medium (DMEM) containing GlutaMAX-I and 1 mM sodium pyruvate supplemented with 10 % fetal bovine serum (FBS), 100 U/ml penicillin, and 100 mg/ml streptomycin at 37 °C in a CO_2_ conditioned, humidified incubator. For the experiments, 4–5 × 10^4^ cells were seeded on glass coverslips on 24-well plates and cultured in DMEM (Gibco, High glucose) with 10 % fetal bovine serum. After 24 h, the cells were transfected with recombinant full-length NPSR1 constructs fused with either red fluorescence protein (NPSR1-A-pDsRed) or green fluorescent protein (NPSR1-B-GFP) using 1.3 μl Fugene HD and 0.5 μg of DNA per well. Construction of the expression vectors has been described earlier [35]. After 24 h, the cells were fixed with 4 % paraformaldehyde in phosphate-buffered saline (PBS) for 5 min. After fixation, the cells were permeabilized with 0.1 % Triton X-100 in PBS for staining with anti-NPSR1-A antibodies, whereas the cells for anti-NPSR1-N staining were left untreated. The cells were incubated in 3 % bovine serum albumin (BSA) in PBS for 30 min to block nonspecific binding of the antibodies. Subsequently, the cells were incubated with primary antibodies for 1 h followed by three washing steps with PBS and staining with secondary anti-mouse antibodies labeled with either green (FITC) or red (TRITC) fluorescent tags for 30 min.

Finally, the nuclei were stained with 40,6-diamino-2-phenylindole (DAPI, Sigma-Aldrich), and the cells were mounted on glass slides with ProLong Gold antifade reagent (Invitrogen).

SH-SY5Y human neuroblastoma cells (ATCC, CRL-2266™) were grown in DMEM-GlutaMAX™-I medium (Invitrogen) supplemented with 10 % fetal calf serum, 100 U/ml penicillin, and 100 U/ml streptomycin at 37 °C in a 5 % CO_2_ humidified incubator. Transfections were done with FuGENE reagent (Roche) according to manufacturer’s protocols. In order to obtain stable clones, SH-SY5Y cells were transfected with NPSR1-A-GFP plasmid and selected with 500 μg/ml G418 for 3 weeks.

### Bacterial lysates

The monoclonal antibodies were analyzed by immunoblotting of recombinant NPSR1 fragments expressed with the pGEX 4 T-3 glutathione-S-transferase (GST) fusion expression vector (Amersham Biosciences, Buckinhamshire, UK) as GST fusion protein in *Escherichia coli* and as a dihydrofolate reductase (DHFR) fusion protein (Qiagen) as described earlier [4]. The constructs for immunoblotting experiments were designed to express the following sequences: CREQRSQDSRMTFRERTER (NPSR1-A) and TEGSFDSSGTGQTLDSSPVA (amino terminus of NPSR1, NPSR1-N).

### Microarray sample preparation and analysis

Cells (2 × 10^6^) were plated, grown O/N, and treated with 100 nM NPS (New England Peptide LLC) for 3 h. Total RNA was extracted using RNeasy® Plus Mini Kit (Qiagen). cDNA was synthesized with TaqMan Reverse Transcription Reagents (Applied Biosystems). A total of 12 hybridizations (technical triplicates) and scannings (Affymetrix GeneChip Scanner 3000) were carried out using standard Affymetrix protocols for gene expression (www.affymetrix.com) with the HGU133plus2 array. Triplicates were analyzed for each of the four samples: untreated parental SHSY5Y and NPSR1-A-GFP1 cells and NPS treated parental and NPSR1-A-GFP1 cells.

### Data analysis for microarrays

Microarray raw data files (.CEL files) were imported into R v. 2.15 (http://cran.r-project.org) and analyzed with the Bioconductor suite v. 2.12 [36]. After quality check operated by affyPLM package [37], the probes were re-annotated and re-assigned to newly-defined probe sets according to the Entrez gene IDs (http://brainarray.mbni.med.umich.edu/Brainarray/Database/CustomCDF/16.0.0/entrezg.asp) and the data were preprocessed with the RMA algorithm [38], as implemented in the Affy package [39]. Subsequently, differential expression was evaluated by fitting a linear model followed by empirical Bayes pairwise comparisons as implemented into the package limma [40]. Genes with *p* < 0.01 after Benjamini and Hochberg post hoc correction [41] were considered to be differentially expressed.

### Quantitative RT-PCR

RNA was extracted from cell lysates using RNeasy®Plus Mini Kit (Qiagen). cDNA was synthesized with TaqMan Reverse Transcription Reagents (Applied Biosystems). Quantitative real-time PCR was done using SYBR Green method with primers as listed in Supplementary Table 1. Quantifications were done using relative standard curve method and the results were normalized with the expression levels of GAPDH. Fold changes were calculated by comparing to the expression level in non-treated cells.

### Statistical analysis

The correlations between marker expression levels and Ki67 PI were analyzed using two-tailed tests and Pearson correlation, *r*. Results are expressed as mean ± SEM and *p* values of <0.05 were considered statistically significant. Statistical analyses were performed using SPSS 17.0 software program (SPSS Inc., Chicago, IL, USA).

## Results

### Specificity of the monoclonal anti-NPSR1 antibodies

Monoclonal antibodies against the N-terminus (anti-NPSR1-N) and C-terminus of NPSR1-A (anti-NPSR1-A) were used to study the expression of NPSR1 in NETs. The specificity of these antibodies has been previously characterized using flow cytometry, immunoblotting experiments, and immunostaining of skin and small intestine sections [7, 30]. In the current study, epitope specificity of the monoclonal anti-NPSR1 antibodies was further studied in immunoblotting experiments of recombinant NPSR1 constructs expressing the corresponding peptide sequences produced in *E. coli* and by immunocytochemistry of the COS-7 cell line transfected with recombinant full-length NPSR1 constructs. Immunoblotting analyses of the bacterial lysates indicate that the monoclonal antibodies against NPSR1-A and NPSR1-N are epitope specific (Supplementary Fig. 1). The correct expression of the recombinant proteins was confirmed through Coomassie Blue staining.

**Fig. 1:**
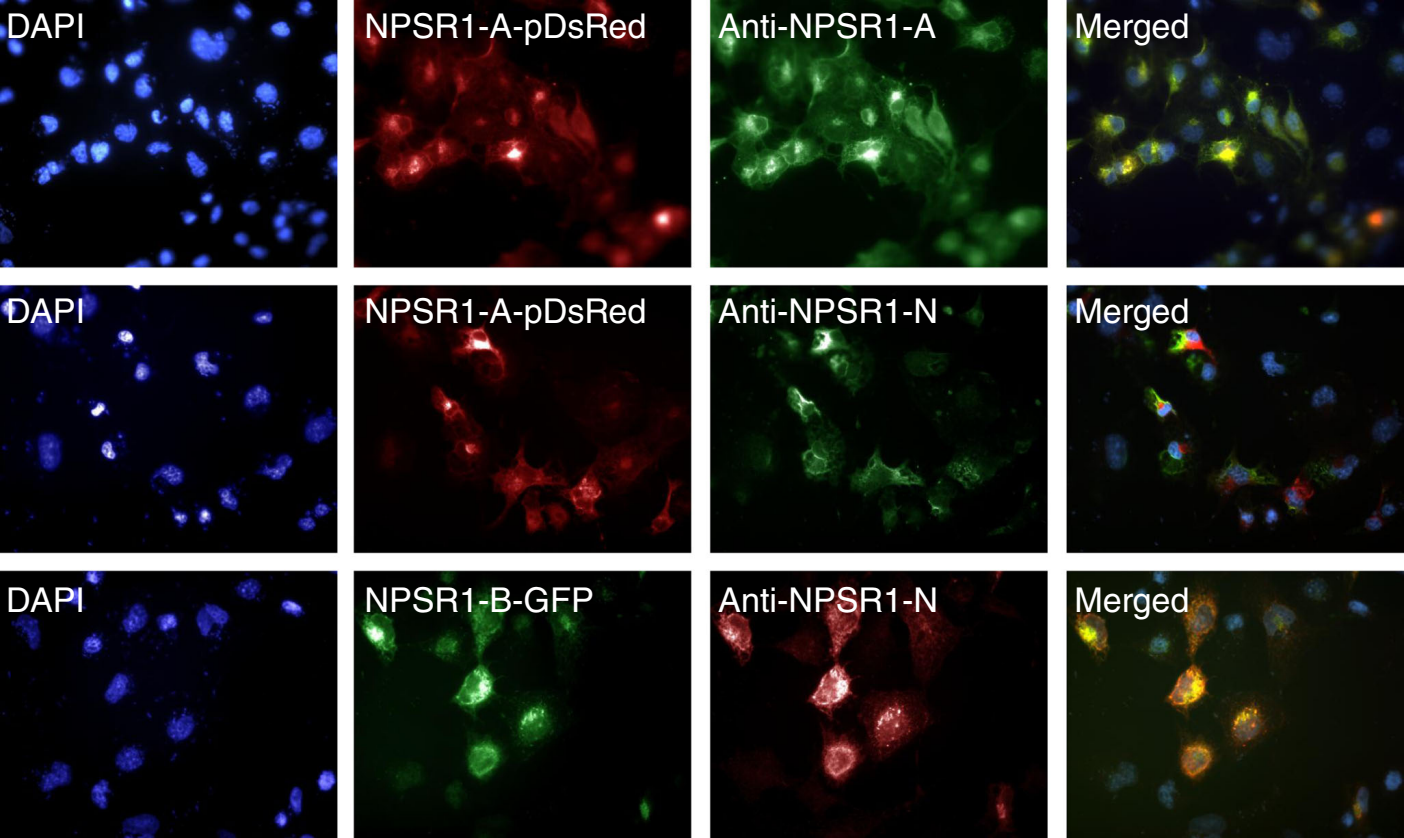
Epitope specificities of the monoclonal anti-NPSR1-A and anti-NPSR1-N (detecting both NPSR1-A and NPSR1-B isoforms) antibodies against recombinant NPSR1 proteins overexpressed in COS-7 cells. The cell nuclei were stained with 40,6-diamino-2-phenylindole (DAPI) (*left panel*). Cells were transfected with the full-length variant for NPSR1-A fused with red fluorescence protein (NPSR1-A-pDsRed) or with the full-length variant for NPSR1-B green fluorescent protein (NPSR1-B-GFP) for 24 h and visualized under conventional fluorescence microscope (*left middle*). Cells transfected with NPSR1-A-pDsRed were stained either with the monoclonal anti-NPSR1-A or anti-NPSR1-N antibodies followed with secondary anti-mouse antibodies labeled with green (FITC) fluorescent tags, and cells transfected with NPSR1-B-GFP were stained with the monoclonal anti-NPSR1-N followed with secondary anti-mouse antibodies labeled with red fluorescent tags (TRITC) (*right middle*). The cells were permeabilized for staining with the anti-NPSR1-A antibodies, whereas the cells for anti-NPSR1-N stainings were left untreated. Overlay of the two images (*right panel*)

Specificity of the monoclonal NPSR1 antibodies in immunocytochemistry was tested on COS-7 cells, transfected with recombinant full-length NPSR1 constructs fused with either red fluorescence protein (NPSR1-A-pDsRed) or green fluorescent protein (NPSR1-B-GFP). Binding of the primary antibodies was visualized using secondary anti-mouse antibodies labeled with either green (FITC) fluorescent tags for NPSR1-A-pDsRed constructs or red (TRITC) fluorescent tags for NPSR1-B-GFP construct. Overlay of the two images (FITC/TRITC) resulted in similar patterns indicating that the antibodies were epitope specific also for the full-length NPSR1 isoforms (Fig. 1).

### NPSR1 is widely expressed in NETs

**Fig. 2:**
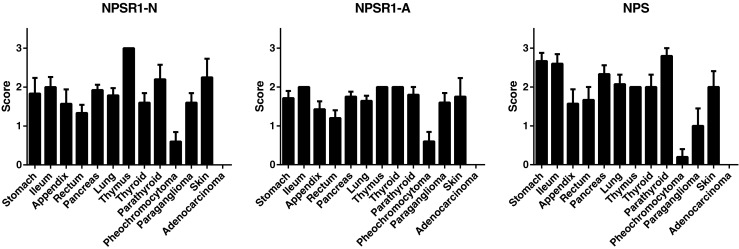
Expression of NPSR1-N, NPSR1-A, and NPS in NETs and colon adenocarcinomas (mean ± SEM)

**Fig. 3:**
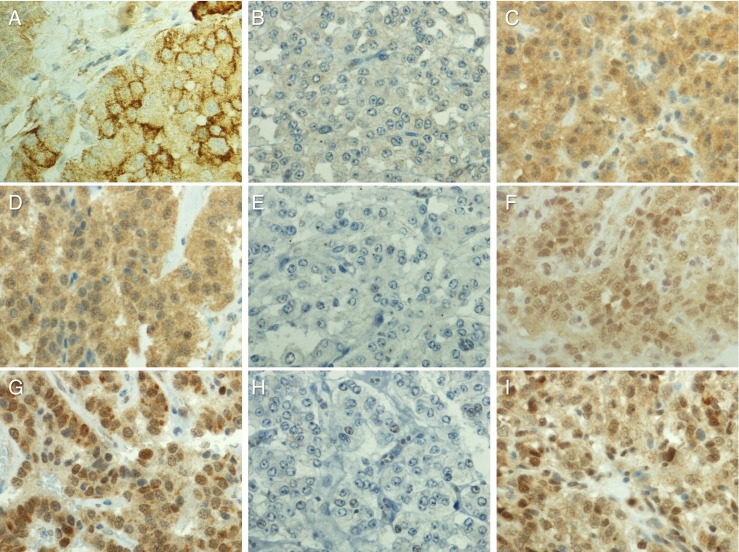
Representative photomicrographs of NPSR1-N (**a**–**c**), NPSR1-A (**d**–**f**), and NPS (**g**–**i**) in NETs from pancreas (**a**, **d**, **g**), pheochromocytoma (**b**, **e**, **h**), and paraganglioma (**c**, **f**, **i**). Original magnification × 600

We have previously shown that NPSR1 is expressed in enteroendocrine cells of the gut [7]. To analyze whether NPS/NPSR1 is a biomarker for NET, NPS and NPSR1 immunoreactivity was assessed on the NET TMA (Table 1). For six samples, no results were obtained either because of missing tumor tissue cores on the TMA or unrepresentative tissue material. NPSR1 and NPS were widely expressed in NETs (Fig. 2). Pheochromocytomas of the adrenal medulla showed no or only very low immunoreactivity for NPSR1 and NPS whereas extra-adrenal sympathetic paragangliomas showed stronger reactivity for the antigens (Fig. 3). Staining with the anti-NPSR1-N antibodies which detect extracellular epitopes of both NPSR1-A and NPSR1-B isoforms resulted in clear staining of the cellular membranes (Fig, 3a) whereas staining with the anti-NPSR1-A antibodies which detect intracellular epitopes of NPSR1-A resulted mostly in cytoplasmic staining. Expression of NPS was mostly cytoplasmic, and some nuclear/perinuclear expression was also observed. Chromogranin-A expression and the Ki-67 proliferation index were inversely correlated (*r* = −0.551, *p* < 0.001).

### Expression of NPSR1 is specific for NETs

To study whether expression of NPSR1 is specific for NETs or a general marker of abnormal growth, we compared the expression of NPSR1 in colorectal adenocarcinomas (*n* = 25) and NETs. As shown in Fig. 2, NPSR1 was not expressed in the adenocarcinoma samples, but was expressed in the NETs of the GI tract (rectum, ileum), suggesting that expression of NPSR1 might be specific for NETs. The colorectal adenocarcinomas did not express the neuroendocrine cell marker chromogranin-A (data not shown).

### NPSR1 expression in metastases

To further assess the impact of NPSR1 expression on metastatic potential, NPSR1 immunoreactivity was analyzed in primary NETs originating from the pancreas and ileum and in tumor metastasis samples from the same tumors (Table 2). We found no difference in expression of NPSR1 between primary NETs and their metastases.

**Table 2:** Metastases

Case		Tissue	PI %	NPSR1-N	NPSR1-A
1	Primary	Pancreas	5	1	1
	Metastase	Liver	4	1	1
2	Primary	Pancreas	1	2	2
	Metastase	Liver	2	2	2
3	Primary	Pancreas	70	3	1
	Metastase	Lymph node	80	2	1
4	Primary	Pancreas	7	2	2
	Metastase	Liver	28	2	2
5	Primary	Ileum	1	2	2
	Metastase	Liver	–	3	2

### NPS induces cancer-related pathways in SH-SY5Y cells over-expressing NPSR1

Endogenous expression of NPSR1 in cultured cells is low with the exception of human Colo205 colon cancer cells [1]. We used human SH-SY5Y neuroblastoma cells, which overexpress NPSR1-A, to study downstream targets of the NPS-NPSR1 signaling pathway by HGU133plus2 array (Affymetrix), which contains over 47,000 probe sets. Cells treated with 100 nM NPS for 3 h were compared with non-treated cells. Additional comparisons were done to parental SH-SY5Y cells with and without NPS treatment. Using cutoff of 1.25- and 0.75-fold changes, 552 and 184 probe sets were found to be up- or down-regulated, respectively, in NPS treated vs. untreated NPSR1-A-GFP overexpressing cells. The genes are listed in Supplementary Material, Supplementary Table 2. NPS treatment induced small changes also in parental SH-SY5Y cells (listed in Supplementary Material, Supplementary Table 3) possibly due to low endogenous expression of NPSR1.

KEGG pathway mapping of the 552 upregulated transcripts found in the comparison of NPS treated vs. non-treated cells showed enrichment of expression of genes of pathways related to the MAPK pathways (with *p* value of 2.3 × 10^−9^), “circadian activity” (*p* = 5.6 × 10^−9^), focal adhesion, transforming growth factor beta (TGFB), and cytokine–cytokine interactions (Supplementary Table 4).

**Fig. 4:**
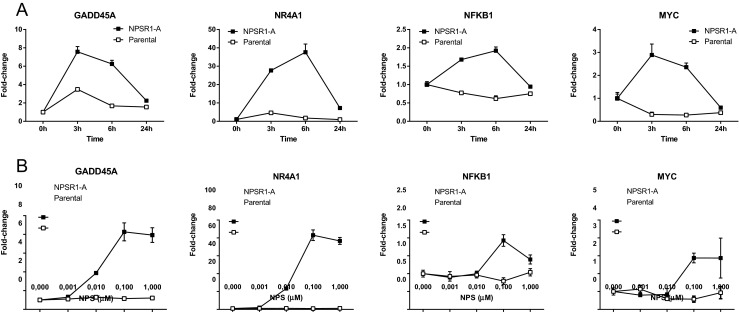
Time and dose course of the gene induction upon NPS treatment. Parental SH-SY5Y cells and cells stably transfected with the NPSR1-A-GFP construct were stimulated **a** with NPS 100 nM for 3–24 h or **b** with 0.001–1 μM NPS for 3 h. mRNA levels of *growth arrest and DNA*-*damage*-*inducible*, *alpha* (*GADD45A*), *nuclear receptor subfamily 4*, *group A*, *member 1* (*NR4A1*), *nuclear factor of kappa light polypeptide gene enhancer in B*-*cells 1* (*NFKB1*), and *v*-*myc avian myelocytomatosis viral oncogene homolog* (*MYC*) were measured by quantitative RT-PCR. The results are shown as average of three biological replicates (time) or three technical replicates (dose) (mean ± SD). The relative change in expression was calculated by comparing to the expression levels in unstimulated cells

To further validate the results, genes that belong to the MAPK pathway were selected from the list and expression was studied by quantitative real-time PCR (qRT-PCR) for the time course and dose responsiveness of the induction. The genes were *growth arrest and DNA*-*damage*-*inducible*, *alpha* (*GADD45A*), *nuclear receptor subfamily 4*, *group A*, *member 1* (*NR4A1*), *nuclear factor of kappa light polypeptide gene enhancer in B*-*cells 1* (*NFKB1*), and *v*-*myc avian myelocytomatosis viral oncogene homolog* (*MYC*). As shown in Fig. 4, expression of *MYC* and *GADD45A* peaked at 3 h, and for *NR4A1* and *NFKB1*, the highest levels were seen at 6 h. Parental SH-SY5Y cell line was used as a control for the experiments.

## Discussion

In this study, we investigated whether NPS or NPSR1 might be used as biomarker for NET in a wide panel of neuroendocrine tumors, characterized by assessing the Ki-67 proliferation index and chromogranin-A and synaptophysin expression. Our results show that NPS and NPSR1 are expressed in NETs. The results from the cell line experiments indicate that stimulation of NPSR1 with NPS results in activation of pathways that are relevant for cancer development.

We used monoclonal antibodies against the N-terminus and C-terminus of NPSR1 to characterize its expression in NETs. In previous studies, we found anti-NPSR1-N antibodies to be specific for HEK-293 cells transiently overexpressing NPSR1-A and NPSR1-B in flow cytometry experiments [30], and anti-NPSR1-A antibodies specific for HEK-293 cells stably overexpressing NPSR1-A in immunoblotting experiments [7]. Furthermore, staining of small intestinal mucosa with monoclonal anti-NPSR1-A antibodies and carefully characterized polyclonal antibodies against NPSR1-A [2, 4, 9] confirmed that NPSR1-A is expressed in the enteroendocrine cells of the gut [7]. Another study confirmed that staining of ileum, duodenum, colon, and rectum samples with publicly available (www.proteinatlas.org) affinity-purified (monospecific) NPSR1-antibodies resulted in strong and specific staining of enteroendocrine cells [8]. The results from the current study confirm the epitope specificity of monoclonal NPSR1 antibodies and the cell surface expression of NPSR1 in NETs.

The WHO classification of GEP-NETs is based on the proliferation rate, and the tumors are categorized into different grades depending on proliferation activity: NET G1 tumors PI = 0–2 % and NET G2 tumors PI = 3–20 %. Neuroendocrine carcinomas (NEC) have a PI over 20 %. Our results show that the expression of NPS/NPSR1 is specific for NETs as NPSR1 was expressed in the NETs of the GI tract but not in colorectal adenocarcinoma samples from the same primary site.

One of our most interesting findings was that NPSR1 is expressed in sympathetic extra adrenal paragangliomas but not in pheochromocytomas arising from the adrenal medulla. Paragangliomas are classified either as parasympathetic or sympathetic paraganglioma depending on their origin. Unlike parasympathetic paragangliomas, pheochromocytomas and sympathetic paragangliomas usually produce catecholamines and are more frequently malignant. Sympathetic paragangliomas metastasize more frequently than pheochromocytomas [42, 43]. Currently, only three predictors of metastatic potential in patients with pheochromocytomas and sympathetic paragangliomas are well recognized: primary tumor location (adrenal pheochromocytomas vs. extra-adrenal sympathetic paragangliomas), primary tumor size, and germline mutations of the SDHB (succinate dehydrogenase complex, subunit B, iron sulfur) gene. More than half of the patients with metastatic sympathetic paraganglioma have an underlying *SDHB* mutation. These mutations are also found in 5–10 % of patients with metastatic pheochromocytomas. As the role of NPS in these tumors appears to be associated with malignant outcome, lack of expression in pheochromocytomas may be related to benign tumor behavior.

In previous studies, NPS stimulation was found to stimulate growth of Colo205 human colon cancer cells, possibly via enhanced phosphorylation of MAPK [1], and provoked proliferation of pig splenic lymphocytes [5]. In an NPSR1 overexpressing human embryonic kidney epithelial cell line, NPS stimulation resulted in decreased cell proliferation [2]. The current grading system for NETs is based on mitotic rate and proliferation index. Ki-67 is an established marker of cell proliferation that is expressed in all stages of the cell cycle except for the resting phase. PI is generally considered to be applicable for low-grade tumors, such as tumors of the GI tract. Although proliferation is an essential marker and determines the prognosis and guides treatment of NETs, it does not necessarily reflect metastatic potential. For example, small bowel NETs may have a very low proliferation and yet be widely metastasized. In our material, we did not find any difference in NPSR1 expression in primary vs. metastatic tumors. NPS might not take part in the processes involved in NET metastasis (vascular invasion, homing and tumor neovascularization) [44].

Signaling pathways downstream of NPSR1 have been extensively investigated using HEK293 cells overexpressing NPSR1 stimulated with 1–2 μM NPS for 6 h [29, 30, 45]. We used SH-SY5Y neuroblastoma cells with the same arrays (HGU133plus2) and the same cutoff (*B* value >7). However, we used 100 nM NPS and 3 h exposure, which allowed us to asses early induced genes. Pathway analysis revealed that MAPK is the most significantly induced signaling pathway that was previously linked with NPSR1. We selected genes that belong to the MAPK pathway and showed that their regulation is dependent on NPS stimulation. The other highly significant gene ontology group induced by NPS was the circadian clock gene pathway. Interestingly, phenotypical analysis of *Npsr1* knockout mice has revealed deficits in circadian activity [46, 47], and central administration of NPS induces wakefulness in various rodent models [3]. Furthermore, polymorphism in NPSR1 was associated with bedtime and sleepiness in a genome-wide association study [48]. Motivated by these findings, we previously showed that NPS affects circadian clock gene expression in the same SH-SY5Y cell line with NPSR1 overexpression [35]. Of these genes that regulate circadian molecular clock machinery, *NPAS2*, *PER1*, *CRY1*, and *RORA* were among the most affected genes in the current microarray experiment. Epidemiological studies have shown that disruption of sleep/circadian control due to jet lag, shift work, or increased exposures to light at night has widespread effects on all aspects of neuroendocrine function including elevated risk for cancer. Furthermore, circadian clock regulates the cell cycle, DNA damage responses, ageing, and metabolism [49]. Aberrant circadian rhythms could lead to defects in the regulation of these processes, which might result in tumorigenesis and tumor progression. In addition, the other pathways affected by NPS stimulation, namely focal adhesion, TGFB, and cytokine–cytokine interactions are highly relevant for tumor progression and metastasis. Assembly of focal adhesion is crucial in cell migration, and previous studies have shown that NPS stimulates human monocyte chemotaxis [6] and eosinophil migration and adhesion [50]. Taken together, transcriptome analysis of NPS/NPSR1 ligand/receptor activation is a powerful tool that revealed novel cancer-related gene pathways many of which have already shown to be relevant for NPS/NPSR1 function in experimental settings.

In conclusion, NPSR1 is a marker widely expressed in NET with the exception of adrenal pheochromocytomas. NPSR1 stimulation activates intracellular pathways relevant for cell growth. No differences were detected between the expression levels in primary tumor tissue and metastases.

## Electronic supplementary material

Below is the link to the electronic supplementary material.

ESM 1 (PDF 331 kb)

ESM 2 (XLSX 12 kb)

ESM 3 (XLSX 75 kb)

ESM 4 (XLSX 70 kb)

ESM 5 (XLSX 16 kb)

## References

[1] Reinscheid RK, Xu YL, Okamura N, Zeng J, Chung S, Pai R, Wang Z, Civelli O (2005). Pharmacological characterization of human and murine neuropeptides receptor variants. J Pharmacol Exp Ther.

[2] Vendelin J, Pulkkinen V, Rehn M, Pirskanen A, Raisanen-Sokolowski A, Laitinen A, Laitinen LA, Kere J, Laitinen T (2005). Characterization of GPRA, a novel G protein-coupled receptor related to asthma. Am J Respir Cell Mol Biol.

[3] Xu YL, Gall CM, Jackson VR, Civelli O, Reinscheid RK (2007). Distribution of neuropeptide S receptor mRNA and neurochemical characteristics of neuropeptide S-expressing neurons in the rat brain. J Comp Neurol.

[4] Pulkkinen V, Majuri ML, Wang G, Holopainen P, Obase Y, Vendelin J, Wolff H, Rytilä P, Laitinen LA, Haahtela T, Laitinen T, Alenius H, Kere J, Rehn M (2006). Neuropeptide S and G protein-coupled receptor 154 modulate macrophage immune responses. Hum Mol Genet.

[5] Yao Y, Su J, Yang G, Zhang G, Lei Z, Zhang F, Li X, Kou R, Liu Y, Liu J (2011). Effects of neuropeptide S on the proliferation of splenic lymphocytes, phagocytosis, and proinflammatory cytokine production of pulmonary alveolar macrophages in the pig. Peptides.

[6] Filaferro M, Novi C, Ruggieri V, Genedani S, Alboni S, Malagoli D, Caló G, Guerrini R, Vitale G (2013). Neuropeptide S stimulates human monocyte chemotaxis via NPS receptor activation. Peptides.

[7] Sundman L, Saarialho-Kere U, Vendelin J, Lindfors K, Assadi G, Kaukinen K, Westerholm-Ormio M, Savilahti E, Mäki M, Alenius H, D'Amato M, Pulkkinen V, Kere J, Saavalainen P (2010). Neuropeptide S receptor 1 expression in the intestine and skin—putative role in peptide hormone secretion. Neurogastroenterol Motil.

[8] Camilleri M, Carlson P, Zinsmeister AR, McKinzie S, Busciglio I, Burton D, Zucchelli M, D'Amato M (2010). Neuropeptide S receptor induces neuropeptide expression and associates with intermediate phenotypes of functional gastrointestinal disorders. Gastroenterology.

[9] Laitinen T, Polvi A, Rydman P, Vendelin J, Pulkkinen V, Salmikangas P, Makela S, Rehn M, Pirskanen A, Rautanen A, Zucchelli M, Gullstén H, Leino M, Alenius H, Petäys T, Haahtela T, Laitinen A, Laprise C, Hudson TJ, Laitinen LA, Kere J (2004). Characterization of a common susceptibility locus for asthma-related traits. Science.

[10] Melen E, Bruce S, Doekes G, Kabesch M, Laitinen T, Lauener R, Lindgren CM, Riedler J, Scheynius A, van Hage-Hamsten M Kere J, Pershagen G, Wickman M, Nyberg F; PARSIFAL Genetics Study Group (2005). Haplotypes of G protein-coupled receptor 154 are associated with childhood allergy and asthma. Am J Respir Crit Care Med.

[11] Kormann M, Carr D, Klopp N, Illig T, Leupold W, Fritzsch C, Weiland S, von Mutius E, Kabesch M (2005). G-protein coupled receptor polymorphisms are associated with asthma in a large German population. Am J Respir Crit Care Med.

[12] Feng Y, Hong X, Wang L, Jiang S, Chen C, Wang B, Yang J, Fang Z, Zang T, Xu X, Xu X (2006). G protein-coupled receptor 154 gene polymorphism is associated with airway hyperresponsiveness to methacholine in a Chinese population. J Allergy Clin Immunol.

[13] Malerba G, Lindgren CM, Xumerle L, Kiviluoma P, Trabetti E, Laitinen T, Galavotti R, Pescollderungg L, Boner AL, Kere J, Pignatti PF (2007). Chromosome 7p linkage and GPR154 gene association in Italian families with allergic asthma. Clin Exp Allergy.

[14] Hersh CP, Raby BA, Soto-Quiros ME, Murphy AJ, Avila L, Lasky-Su J, Sylvia JS, Klanderman BJ, Lange C, Weiss ST, Celedón JC (2007). Comprehensive testing of positionally cloned asthma genes in two populations. Am J Respir Crit Care Med.

[15] Vergara C, Jimenez S, Acevedo N, Martinez B, Mercado D, Gusmao L, Rafaels N, Hand T, Barnes KC, Caraballo L (2009). Association of G-protein-coupled receptor 154 with asthma and total IgE in a population of the Caribbean coast of Colombia. Clin Exp Allergy.

[16] Castro-Giner F, de Cid R, Gonzalez JR, Jarvis D, Heinrich J, Janson C, Omenaas ER, Matheson MC, Pin I, Anto JM, Wjst M, Estivill X, Kogevinas M (2010). Positionally cloned genes and age-specific effects in asthma and atopy: an international population-based cohort study (ECRHS). Thorax.

[17] D'Amato M, Bruce S, Bresso F, Zucchelli M, Ezer S, Pulkkinen V, Lindgren C, Astegiano M, Rizzetto M, Gionchetti P, Riegler G, Sostegni R, Daperno M, D'Alfonso S, Momigliano-Richiardi P, Torkvist L, Puolakkainen P, Lappalainen M, Paavola-Sakki P, Halme L, Farkkila M, Turunen U, Kontula K, Lofberg R, Pettersson S, Kere J (2007). Neuropeptide s receptor 1 gene polymorphism is associated with susceptibility to inflammatory bowel disease. Gastroenterology.

[18] D'Amato M, Zucchelli M, Seddighzadeh M, Anedda F, Lindblad S, Kere J, Alfredsson L, Klareskog L, Padyukov L (2010). Analysis of neuropeptide S receptor gene (NPSR1) polymorphism in rheumatoid arthritis. PLoS One.

[19] Robledo G, Gonzalez-Gay MA, Fernandez-Gutierrez B, Lamas JR, Balsa A, Pascual-Salcedo D, Castaneda S, Blanco R, Gonzalez-Alvaro I, Garcia A, Raya E, Gómez-Vaquero C, Delgado M, Martín J (2012). NPSR1 gene is associated with reduced risk of rheumatoid arthritis. J Rheumatol.

[20] Donner J, Haapakoski R, Ezer S, Melen E, Pirkola S, Gratacos M, Zucchelli M, Anedda F, Johansson LE, Soderhall C, Orsmark-Pietras C, Suvisaari J, Martín-Santos R, Torrens M, Silander K, Terwilliger JD, Wickman M, Pershagen G, Lönnqvist J, Peltonen L, Estivill X, D'Amato M, Kere J, Alenius H, Hovatta I (2010). Assessment of the neuropeptide S system in anxiety disorders. Biol Psychiatry.

[21] Okamura N, Hashimoto K, Iyo M, Shimizu E, Dempfle A, Friedel S, Reinscheid RK (2007). Gender-specific association of a functional coding polymorphism in the Neuropeptide S receptor gene with panic disorder but not with schizophrenia or attention-deficit/hyperactivity disorder. Prog Neuro-Psychopharmacol Biol Psychiatry.

[22] Raczka KA, Gartmann N, Mechias ML, Reif A, Büchel C, Deckert J, Kalisch R (2010). A neuropeptide S receptor variant associated with overinterpretation of fear reactions: a potential neurogenetic basis for catastrophizing. Mol Psychiatry.

[23] Domschke K, Reif A, Weber H, Richter J, Hohoff C, Ohrmann P, Pedersen A, Bauer J, Suslow T, Kugel H, Heindel W, Baumann C, Klauke B, Jacob C, Maier W, Fritze J, Bandelow B, Krakowitzky P, Rothermundt M, Erhardt A, Binder EB, Holsboer F, Gerlach AL, Kircher T, Lang T, Alpers GW, Ströhle A, Fehm L, Gloster AT, Wittchen HU, Arolt V, Pauli P, Hamm A, Deckert J (2011). Neuropeptide S receptor gene —converging evidence for a role in panic disorder. Mol Psychiatry.

[24] Kumsta R, Chen FS, Pape HC, Heinrichs M (2013). Neuropeptide S receptor gene is associated with cortisol responses to social stress in humans. Biol Psychiatry.

[25] Xu YL, Reinscheid RK, Huitron-Resendiz S, Clark SD, Wang Z, Lin SH, Brucher FA, Zeng J, Ly NK, Henriksen SJ, de Lecea L, Civelli O (2004). Neuropeptide S: a neuropeptide promoting arousal and anxiolytic-like effects. Neuron.

[26] Okamura N, Reinscheid RK (2007). Neuropeptide S: a novel modulator of stress and arousal. Stress.

[27] Zhu H, Mingler MK, McBride ML, Murphy AJ, Valenzuela DM, Yancopoulos GD, Williams MT, Vorhees CV, Rothenberg ME (2010). Abnormal response to stress and impaired NPS-induced hyperlocomotion, anxiolytic effect and corticosterone increase in mice lacking NPSR1. Psychoneuroendocrinology.

[28] Jüngling K, Liu X, Lesting J, Coulon P, Sosulina L, Reinscheid RK, Pape HC (2012). Activation of neuropeptide S-expressing neurons in the locus coeruleus by corticotropin-releasing factor. J Physiol.

[29] Vendelin J, Bruce S, Holopainen P, Pulkkinen V, Rytilä P, Pirskanen A, Rehn M, Laitinen T, Laitinen LA, Haahtela T, Saarialho-Kere U, Laitinen A, Kere J (2006). Downstream target genes of the neuropeptide S-NPSR1 pathway. Hum Mol Genet.

[30] Pietras CO, Vendelin J, Anedda F, Bruce S, Adner M, Sundman L, Pulkkinen V, Alenius H, D'Amato M, Söderhäll C, Kere J (2011). The asthma candidate gene NPSR1 mediates isoform specific downstream signalling. BMC Pulm Med.

[31] Kloppel G (2007). Tumour biology and histopathology of neuroendocrine tumours. Best Pract Res Clin Endocrinol Metab.

[32] Koskensalo S, Louhimo J, Hagström J, Lundin M, Stenman UH, Haglund C (2013). Concomitant tumor expression of EGFR and TATI/SPINK1 associates with better prognosis in colorectal cancer. PLoS One.

[33] Remes SM, Tuominen VJ, Helin H, Isola J, Arola J (2012). Grading of neuroendocrine tumors with Ki-67 requires high-quality assessment practices. Am J Surg Pathol.

[34] Tuominen VJ, Ruotoistenmäki S, Viitanen A, Jumppanen M, Isola J (2010). ImmunoRatio: a publicly available web application for quantitative image analysis of estrogen receptor (ER), progesterone receptor (PR), and Ki-67. Breast Cancer Res.

[35] Acevedo N, Sääf A, Söderhäll C, Melén E, Mandelin J, Pietras CO, Ezer S, Karisola P, Vendelin J, Gennäs GB, Yli-Kauhaluoma J, Alenius H, von Mutius E, Doekes G, Braun-Fahrländer C, Riedler J, van Hage M, D'Amato M, Scheynius A, Pershagen G, Kere J, Pulkkinen V (2013). Interaction of retinoid acid receptor-related orphan receptor alpha (RORA) and neuropeptide S receptor 1 (NPSR1) in asthma. PLoS One.

[36] Gentleman RC, Carey VJ, Bates DM, Bolstad B, Dettling M, Dudoit S, Ellis B, Gautier L, Ge Y, Gentry J, Hornik K, Hothorn T, Huber W, Iacus S, Irizarry R, Leisch F, Li C, Maechler M, Rossini AJ, Sawitzki G, Smith C, Smyth G, Tierney L, Yang JY, Zhang J (2003). Bioconductor: open software development for computational biology and bioinformatics. Genome Biol.

[37] Brettschneider J, Collin F, Bolstad BM, Speed TP (2008). Quality assessment for short oligonucleotide microarray data. Technometrics.

[38] Irizarry RA, Hobbs B, Collin F, Beazer-Barclay YD, Antonellis KJ, Scherf U, Speed TP (2003). Exploration, normalization, and summaries of high density oligonucleotide array probe level data. Biostatistics.

[39] Gautier L, Cope LM, Bolstad BM, Irizarry RA (2004). affy—analysis of Affymetrix GeneChip data at the probe level. Bioinformatics.

[40] Smyth GK (2005) Limma: linear models for microarray data. In: Gentleman R, Carey V, Dudoit S, Irizarry R, Huber W (eds) Bioinformatics and computational biology solutions using R and bioconductor. Springer, New York, p 397–420

[41] Benjamini Y, Hochberg Y (1995). Controlling the false discovery rate: a practical and powerful approach to multiple testing. J Royal Stat Soc Series B (Methodological).

[42] Ayala-Ramirez M, Feng L, Johnson MM, Ejaz S, Habra MA, Rich T, Busaidy N, Cote GJ, Perrier N, Phan A, Patel S, Waguespack S, Jimenez C (2011). Clinical risk factors for malignancy and overall survival in patients with pheochromocytomas and sympathetic paragangliomas: primary tumor size and primary tumor location as prognostic indicators. J Clin Endocrinol Metab.

[43] Jimenez C, Rohren E, Habra MA, Rich T, Jimenez P, Ayala-Ramirez M, Baudin E (2013). Current and future treatments for malignant pheochromocytoma and sympathetic paraganglioma. Curr Oncol Rep.

[44] Hanahan D, Weinberg RA (2000). The hallmarks of cancer. Cell.

[45] Anedda F, Zucchelli M, Schepis D, Hellquist A, Corrado L, D'Alfonso S, Achour A, McInerney G, Bertorello A, Lördal M, Befrits R, Björk J, Bresso F, Törkvist L, Halfvarson J, Kere J, D'Amato M (2011). Multiple polymorphisms affect expression and function of the neuropeptide S receptor (NPSR1). PLoS One.

[46] Duangdao DM, Clark SD, Okamura N, Reinscheid RK (2009). Behavioral phenotyping of neuropeptide S receptor knockout mice. Behav Brain Res.

[47] Fendt M, Buchi M, Burki H, Imobersteg S, Ricoux B, Suply T, Sailer AW (2011). Neuropeptide S receptor deficiency modulates spontaneous locomotor activity and the acoustic startle response. Behav Brain Res.

[48] Gottlieb DJ, O'Connor GT, Wilk JB (2007). Genome-wide association of sleep and circadian phenotypes. BMC Med Genet.

[49] Fu L, Kettner NM (2013). The circadian clock in cancer development and therapy. Prog Mol Biol Trans Sci.

[50] Ilmarinen P, James A, Moilanen E, Pulkkinen V, Daham K, Saarelainen S, Laitinen T, Dahlén SE, Kere J, Dahlén B, Kankaanranta H (2014). Enhanced expression of neuropeptide S (NPS) receptor in eosinophils from severe asthmatics and subjects with total IgE above 100 IU/ml. Peptides.

