# Supplementary material for: Neuropeptide S receptor 1 (NPSR1) activates cancer-related pathways and is widely expressed in neuroendocrine tumors
Source: Virchows Arch. 2014 Jun 12;465(2):173–83. doi: 10.1007/s00428-014-1602-x (PMC4116602; doi:10.1007/s00428-014-1602-x)
Supplement: Supplementary file 1 — (PDF 331 kb) [file 428_2014_1602_MOESM1_ESM.pdf]

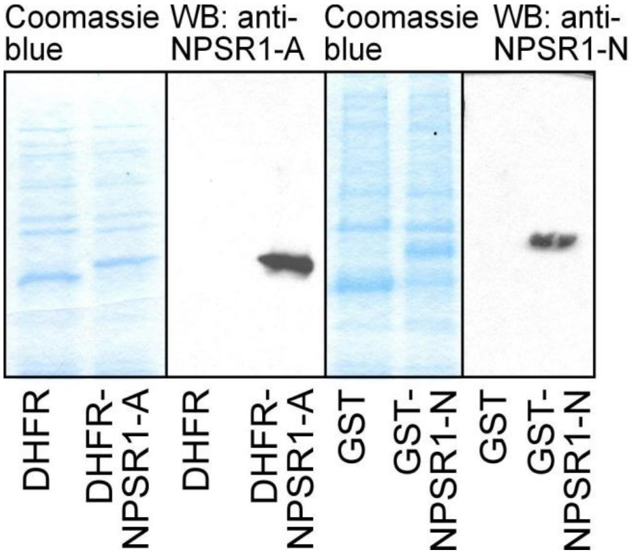

Supplementary Figure 1. Epitope specificities of the monoclonal anti-NPSR1 antibodies against the recombinant NPSR1 constructs produced in *E. coli*. The constructs were designed to express the following sequences: CREQRSQDSRMTFRERTER (NPSR1-A) and TEGSFDSSGTGQTLDSSPVA (amino terminus of NPSR1, NPSR1-N). The correct expression of the recombinant proteins was confirmed with Coomassie Blue stainings. Immunoblotting analyses of the bacterial lysates indicate that the monoclonal antibodies against NPSR1-A and NPSR1-N were epitope specific.
